# Common ground on immune infiltration landscape and diagnostic biomarkers in diabetes-complicated atherosclerosis: an integrated bioinformatics analysis

**DOI:** 10.3389/fendo.2024.1381229

**Published:** 2024-07-31

**Authors:** Yifei Qi, Yan Zhang, Shuang Guan, Li Liu, Hongqin Wang, Yao Chen, Qingbing Zhou, Fengqin Xu, Ying Zhang

**Affiliations:** ^1^ Department of General Medicine, Xiyuan Hospital, China Academy of Chinese Medical Sciences, Beijing, China; ^2^ Institute of Geriatrics, Xiyuan Hospital, China Academy of Chinese Medical Sciences, Beijing, China; ^3^ Institute of Basic Research in Clinical Medicine, China Academy of Chinese Medical Sciences, Beijing, China; ^4^ Innovation Research Institute of Traditional Chinese Medicine, Shandong University of Traditional Chinese Medicine, Jinan, China

**Keywords:** atherosclerosis, type 2 diabetes mellitus, bioinformatics, immune infiltration, molecular mechanisms

## Abstract

**Introduction:**

Type 2 diabetes mellitus (T2DM) is a major cause of atherosclerosis (AS). However, definitive evidence regarding the common molecular mechanisms underlying these two diseases are lacking. This study aimed to investigate the mechanisms underlying the association between T2DM and AS.

**Methods:**

The gene expression profiles of T2DM (GSE159984) and AS (GSE100927) were obtained from the Gene Expression Omnibus, after which overlapping differentially expressed gene identification, bioinformatics enrichment analyses, protein–protein interaction network construction, and core genes identification were performed. We confirmed the discriminatory capacity of core genes using receiver operating curve analysis. We further identified transcription factors using TRRUST database to build a transcription factor–mRNA regulatory network. Finally, the immune infiltration and the correlation between core genes and differential infiltrating immune cells were analyzed.

**Results:**

A total of 27 overlapping differentially expressed genes were identified under the two-stress conditions. Functional analyses revealed that immune responses and transcriptional regulation may be involved in the potential pathogenesis. After protein–protein interaction network deconstruction, external datasets, and qRT-PCR experimental validation, four core genes (IL1B, C1QA, CCR5, and MSR1) were identified. ROC analysis further showed the reliable value of these core genes. Four common differential infiltrating immune cells (B cells, CD4+ T cells, regulatory T cells, and M2 macrophages) between T2DM and AS datasets were selected based on immune cell infiltration. A significant correlation between core genes and common differential immune cells. Additionally, five transcription factors (RELA, NFκB1, JUN, YY1, and SPI1) regulating the transcription of core genes were mined using upstream gene regulator analysis.

**Discussion:**

In this study, common target genes and co-immune infiltration landscapes were identified between T2DM and AS. The relationship among five transcription factors, four core genes, and four immune cells profiles may be crucial to understanding T2DM complicated with AS pathogenesis and therapeutic direction.

## 1 Introduction

Atherosclerosis (AS) is a fatal complication of diabetes mellitus and the leading cause of death for patients worldwide (1). In 2021, there were approximately 537 million people with diabetes worldwide, and patients with type 2 diabetes mellitus (T2DM) represent over 90% of this population. Recent data have suggested that patients with T2DM have advanced coronary plaques with larger necrotic core areas and higher arterial media calcification (2). Severe and extensive AS develops almost two decades earlier than people without T2DM (3). Therefore, to reduce cardiovascular events, improving the diagnosis and treatment of high-risk plaques in susceptible populations is essential (4).

T2DM and AS are chronic inflammatory diseases primarily caused by metabolic disorders. Hyperinsulinemia increases the circulating fat levels of pro-inflammatory and pro-atherogenic factors (5). Similarly, glucose overload induces oxidative stress and activates pro-inflammatory signaling pathways (6). In addition, metabolic disorders are associated with an altered immune response. Autoimmunity is crucial in the coronary artery formation process, such as fat streak formation, plaque calcification, plaque rupture, and thrombosis.

Although these factors contribute to the modification of microvascular and macrovascular structures and plaque formation (7), the systematic pathological mechanism of T2DM complicated with AS in a genetic and cellular level is still unclear. Therefore, it restricts the research and development of targeted drugs. Bioinformatics analysis based on high-throughput data and gene microarray technology have provided new strategies for discovering therapeutic targets in recent years. We obtain high-throughput data and microarray datasets from GEO to investigate overlapping differentially expressed gene (DEGs) between T2DM and AS. Then, the network deconstruction method was used to dimension reduction to obtain the core genes. The biological function of the core genes were determined by enrichment analysis, and the correlation between core genes and the differential infiltrated immune cells (IICs), which was screened by immune infiltration analysis, was confirmed by Spearman test. To sum up, IL1B, C1QA, CCR5, and MSR1 were identified as core genes that might serve as biomarkers for T2DM complicated with AS. They were very informative for diagnosis and may become new therapeutic targets for therapy.

## 2 Materials and methods

### 2.1 Data collection

We searched for related gene expression or high-throughput sequencing datasets using 1) “atherosclerotic” and “type 2 diabetes” as keywords, 2) the test specimens in datasets derived from human tissues, and 3) the largest possible sample size. Finally, two high-throughput sequencing datasets (GSE159984 and GSE164416) and two microarray datasets (GSE100927 and GSE28829) were obtained from the National Center for Biotechnological Information (NCBI) Gene Expression Omnibus https://www.ncbi.nlm.nih.gov/geo/) database. GSE159984 (including 28 patients with T2DM and 58 controls) and GSE100927 (comprising 29 patients with AS and 12 controls) were used to screen for DEGs, while GSE164416 (including 39 patients with T2DM and 18 controls) and GSE28829 (comprising 16 patients with advanced carotid plaque and 13 with early carotid plaque) were used as external validation datasets. **Table 1** summarizes the information for the datasets selected.

**Table 1:** The information of GEO datasets.

GEO dataset	Type	Platform	Disease samples	Sample type in patients	Control samples	Sample type in controls
GSE159984	high throughput sequencing	GPL16791	28	human islets from type 2 diabetic donor	58	human islets from non-diabetic donor
GSE100927	array	GPL17077	29	human atherosclerotic carotid artery from donor	12	human carotid artery arteries without atherosclerotic lesions from control donor
GSE164416	high throughput sequencing	GPL16791	39	human islets from type 2 diabetic donor	18	human islets from non-diabetic donor
GSE28829	array	GPL570	16	advanced atherosclerotic plaque	13	early atherosclerotic plaque

### 2.2 Overlapping DEG identfication and enrichment analyses

DEG analysis was filtered using the “edgeR” or “limma” package (8, 9) in R (version 4.3.1), and the results were visualized using the “ggplot2” package. We obtained DEGs with |log2fold change≥1 and *p*.adj<0.05 in T2DM and AS diseases, respectively. Overlapping DEGs in the same direction between T2DM and AS were identified using the online Venn diagram tool (https://bioinfogp.cnb.csic.es/tools/venny/). KEGG and GO enrichment analyses of the overlapping DEGs were performed using the “clusterProfiler” package (10).

### 2.3 Protein–protein interaction network construction and hub gene screening

The overlapping genes were imported into the STRING database (http://string-db.org) (11) to construct a protein–protein interaction (PPI) network with complex relationships (interactions combined score >0.4), and this network was visualized in Cytoscape 3.82 (version 3.8.1). Four algorithms (MCC, MNC, Degree, and Closeness) take intersection to identify hub genes using the cytoHubba plug-in (12). Enrichment analysis and co-expression networks of hub genes were performed using “clusterProfiler” package and GeneMANIA (http://www.genemania.org/) (13), respectively. The molecular complex detection technology (MCODE), a plugin in Cytoscape, was used to deconstruct the functional modules. The selection criteria were set as degree cutoff=2, K-core=2, node score cutoff=0.2, and maximum depth=100.

### 2.4 External database verification and qRT-PCR experiments analysis

The GSE164416 and GSE28829 datasets were used for external verification. Significance was calculated using the Student’s t-test, and *p* < 0.05 was considered statistically significant. Then, statistically significant genes were selected for further quantitative real-time PCR (qRT-PCR) analysis in animal models. Male C57BL/6J and ApoE^−/−^ mice were purchased from Weitonglihua Corporation (Beijing, China). To induce diabetic atherosclerosis models, ApoE^−/−^ mice were given streptozocin (50 mg/kg/day) by intraperitoneal injection for 5 days consecutively and fed on high-fat diet for 16 weeks (14). C57BL/6J mice were fed on normal-chow diet for 16 weeks after injected with vehicle used in the control group, pool of two groups, five mice per group. The total RNA of thoracic aortas was isolated with RNA extraction reagent (G3013, Servicebio, China) and reverse transcribed with SweScript All-in-one RT SuperMix (G3337, Servicebio, China). Reactions were run using CFX Connect (Bio-Rad) with 2× Universal Blue SYBR Green qPCR Master Mix (G3326, Servicebio, China). GAPDH was deemed as an internal control, and the results were determined with the 2^−ΔΔCt^ method.

Similarly, the level of significance used was 0.05.

Primers sequences are listed in **Supplementary Table S1**. All animal care and experimental procedures were approved by the animal ethics committee of the Ethics Committee of Xiyuan Hospital, China Academy of Chinese Medical Sciences.

### 2.5 Core gene identification and diagnosing

Based on the results of qRT-PCR analysis, genes with significant statistical differences (*p* < 0.05) were regarded as core genes. Thus, core genes were identified successfully through multiple bioinformatics mining, external datasets validation, and qRT-PCR experimental verification. To determine the value of each core genes and multiple genes in diagnosis of T2DM complicated with AS, we executed receiver operating characteristic (ROC) curve analysis, respectively. The diagnostic capacity of core genes was quantified using the area under ROC curve (AUC) in GSE164416 and GSE28829 datasets. The “pROC” R package was used to generate ROC curves (15). The greater the AUC value, the more superior the discriminatory ability of the model. An AUC closer to 1 indicates better prediction, and an AUC>0.7 indicates good diagnostic efficacy.

### 2.6 Transcription factor prediction

Transcriptional regulatory relationships unraveled by sentence-based text mining (TRRUST) were used to obtain candidate transcription factors (TFs) that regulate core genes (16). This database contains abundant information about TFs associated with target genes and their regulatory relationships with TFs. Statistical significance was defined as an adjusted *p*<0.05. We constructed a TF mRNA regulatory network and visualized it using Cytoscape.

### 2.7 Immune cell infiltration calculation and correlation analysis

We performed immune infiltration analysis to reveal the underlying immune pathogenesis of T2DM complicated with AS. QuanTIseq is a validated deconvolution-based algorithm that estimates the absolute proportions of relevant immune cell types. Thus, we used quanTIseq algorithm to obtain the immune cell infiltration differences between normal group and disease group. Based on “IOBR” packages, relative percentage, different immune cell types were analyzed using GSE164416 and GSE100927 datasets. The results were output as bar graphs and violon plots, respectively. Subsequently, the correlations between core genes and IICs were conducted using Spearman analysis, and the results were visualized using “ggplot2” package.

## 3 Results

### 3.1 Identification and functional analysis of overlapping DEGs

**Figure 1:**
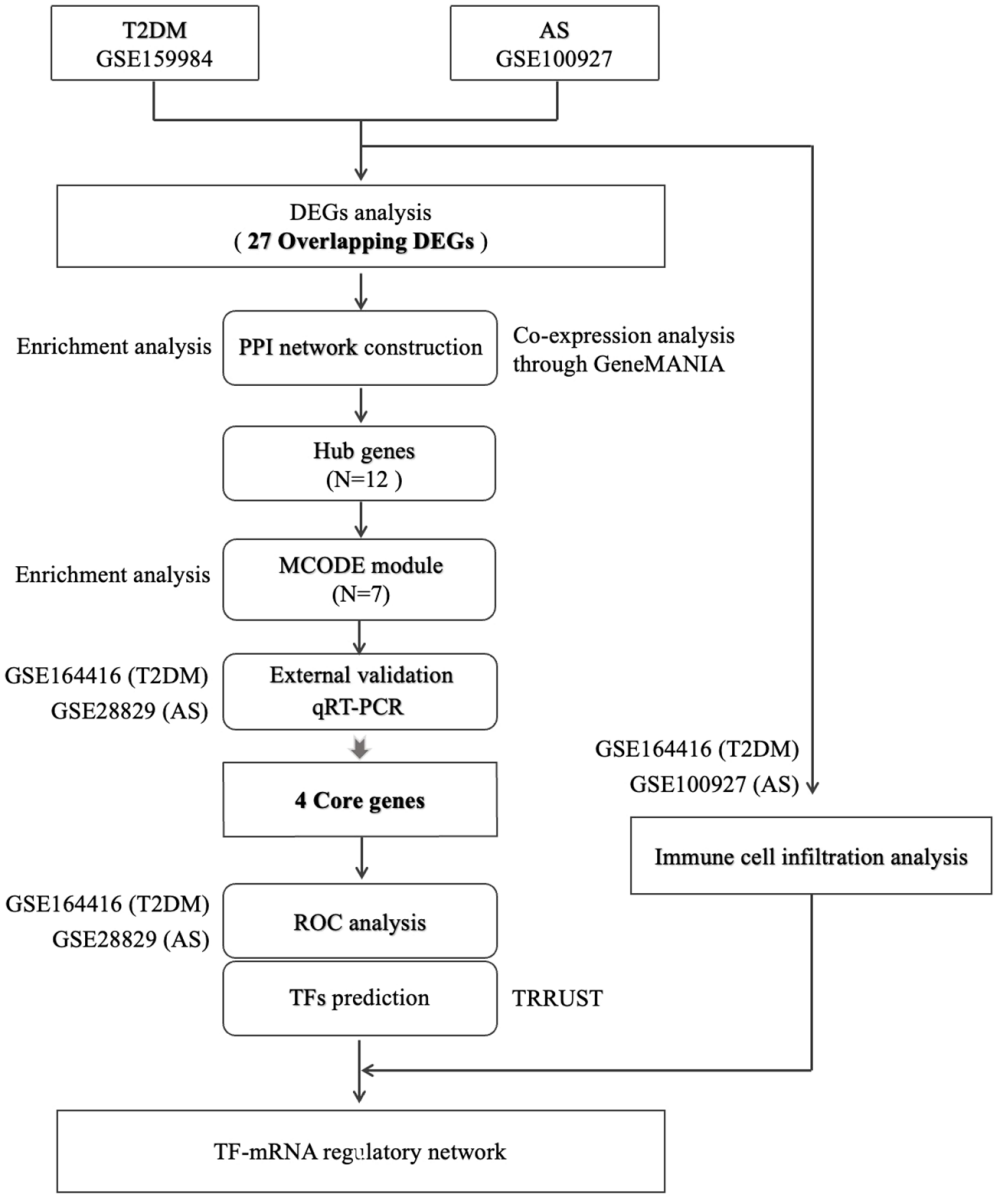
Flow diagram for this study.

**Figure 2:**
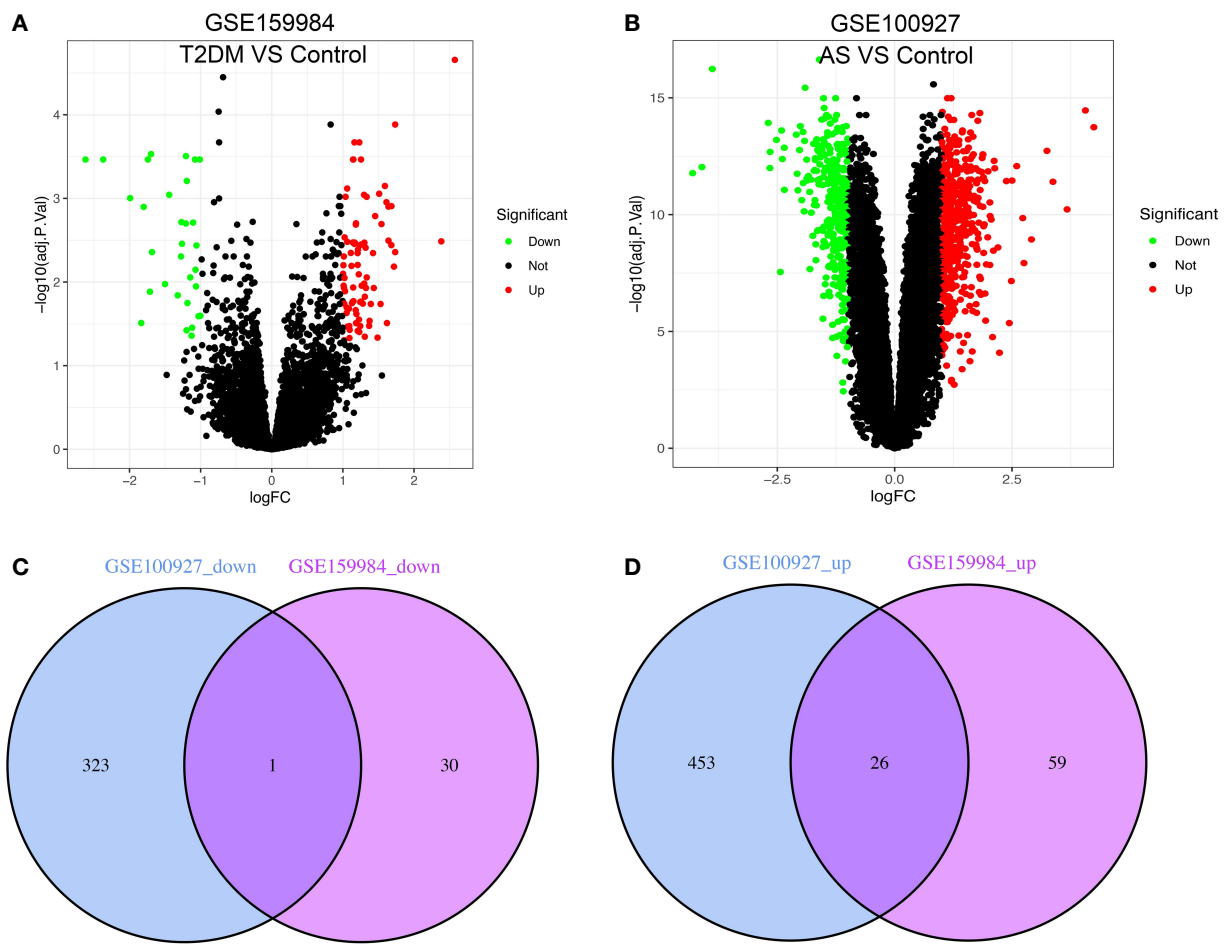
Venn diagram shows the intersection of differentially expressed genes (DEGs) between type 2 diabetes mellitus (T2DM) and atherosclerosis (AS). The volcano plot of the DEGs in GSE159984 and GSE100927 is shown in panels **(A, B)**. Red indicates gene upregulation, green indicates gene downregulation, and gray indicates that the genes had no significant changes. **(C)** Venn diagram shows the downregulated genes in GSE159984 and GSE100927. **(D)** Venn diagram shows the upregulated genes in GSE159984 and GSE100927.

The study’s flow chart is shown in **Figure 1**. In total, 116 and 803 DEGs were obtained from the GSE159984 and GSE100927 datasets, respectively (**Figures 2A, B**). After taking the intersection between the two datasets, 27 overlapping DEGs with the same expression trends (26 upregulated and 1 downregulated) were identified from the two datasets (**Figures 2C, D**). The list of the differential gene expression is included as **Supplementary Data**. The DEGs lists have been included as **Supplementary Tables 2**–**4**.

**Figure 3:**
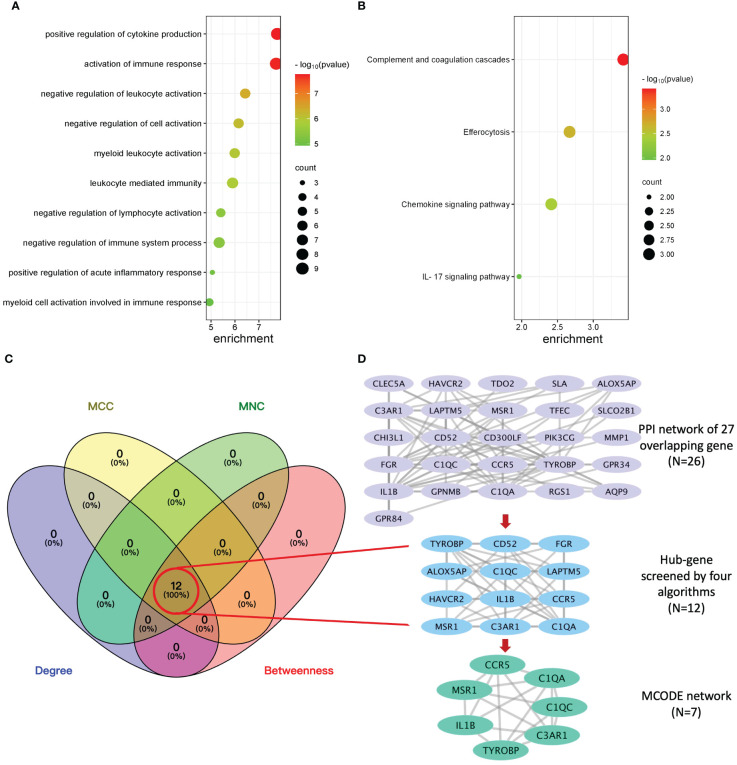
Enrichment analysis, protein–protein interaction (PPI) network, and sub-network construction of overlapping DEGs between T2DM and AS. **(A)** The GO enrichment analysis of the overlapping DEGs. **(B)** The pathway enrichment analysis of the overlapping DEGs. **(C)** The number of screened genes from four algorithms (MCC, MNC, Degree, and Closeness) in the PPI network was indicated as Venn diagrams. **(D)** The results of the PPI network of overlapping genes and sub-network obtained from it. From top to bottom is the overlapping genes’ PPI network, hub genes’ network, and MCODE module network, respectively.

To explore the potential biological function, GO enrichment and KEGG pathway for the overlapping DEGs were performed using R. According to the GO analysis findings, positive regulation of cytokine production, activation of immune response, and negative regulation of leukocyte activation were significantly enriched in the biological process (BP) entries, specific granule, collagen trimer, and specific granule membrane were significantly enriched in the cellular component (CC) entries, and oxidoreductase activity, growth factor receptor binding, and phosphotyrosine residue binding were significantly enriched in the molecular function (MF) entries (**Figure 3A**; **Supplementary Table S5**). In addition, complement and coagulation cascades, chemokine signaling pathway, and IL-17 signaling pathway were significantly enriched in the KEGG entries (**Figure 3B**; **Supplementary Table S6**). These results indicate that immune-related processes and chemokines may be crucial in the development of T2DM complicated with AS.

### 3.2 PPI network construction and hub genes selection

The PPI network of overlapping DEGs contained 26 nodes and 77 interaction pairs. The topological parameters of nodes in this network were are in **Supplementary Table S7**. Four algorithms (MCC, MNC, Degree, and Closeness) in the CytoHubba plugin were used to identify the hub genes (**Supplementary Table S8**). The middle part of **Figure 3C** represents the intersection of the four algorithms. The schematic diagram of PPI network deconstruction and the network constructed with hub genes is shown in **Figure 3D**. A total of 12 hub genes were identified, and these genes were TYROBP, IL1B, C1QA, C3AR1, FGR, CCR5, LAPTM5, C1QC, CD52, MSR1, HAVCR2, and ALOX5AP. To further investigate the biological characteristics of these genes, we analyzed the related functions of hub genes’ co-expression network using GeneMANIA database. The biological functions are associated with immune and inflammatory-related processes, such as the regulation of humoral immune response, complement activation, humoral immune response, interleukin-2 production, and negative regulation of immune system process (**Supplementary Figure S1**).

### 3.3 MCODE module partition and analysis

**Figure 4:**
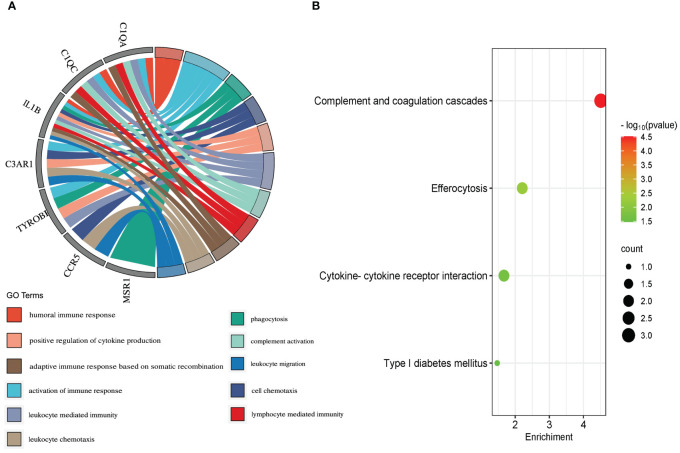
Biological process **(A)** and KEGG pathway analysis **(B)** of the genes in MCODE module.

Molecular complex detection (MCODE) plugin was used to screen out an important subnetwork in hub genes network, and this subnetwork included 7 nodes and 18 pairs. The seven nodes were TYROBP, IL1B, C1QA, C3AR1, CCR5, C1QC, and MSR1, and the scores are shown in **Supplementary Table S9**. Then, the enrichment analysis revealed that the nodes in MCODE network were significantly enriched in inflammatory response and cytokine transcript regulation, specifically, the results of BP enrichment in activation of immune response, leukocyte-mediated immunity, and positive regulation of cytokine production, and the KEGG pathway enrichment in complement and coagulation cascades, efferocytosis, cytokine–cytokine receptor interaction, and type I diabetes mellitus (**Figure 4**; **Supplementary Tables S10**, **S11**). Together with the preceding results, we suggest that changes in the immune microenvironment affected by cytokines and inflammatory responses may be a common mechanism of the T2DM complicated with AS.

### 3.4 Diagnostic efficacy of core genes

**Figure 5:**
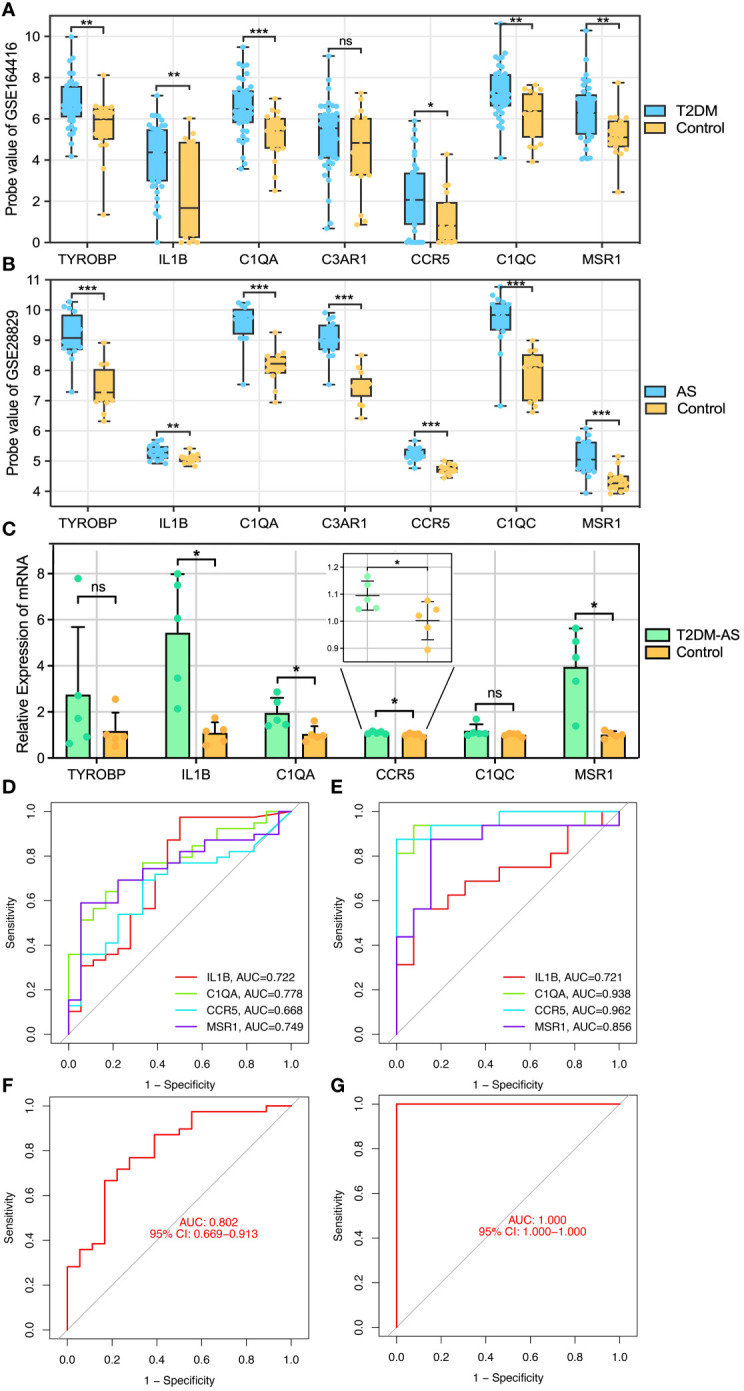
Core genes validation and diagnosis. **(A, B)** External validation: the genes expression in GSE164416 and GSE28829. **(C)** qRT-PCR experimental validation: the genes expression in model mice and control group. Receiver operating curve (ROC) for core gene in **(D)** GSE164416 and **(E)** GSE28829, respectively. Multi-index (all core genes) combined diagnosis in **(F)** GSE164416 and **(G)** GSE28829, respectively. *P < 0.05, **P < 0.01, ***P < 0.001. ns, not statistically significant.

To identify reliable core genes, we carried out both external validation and animal experiments according to genes in MCODE network. The expression levels of seven genes, except for C3AR1, in the T2DM dataset (GSE164416) were significantly higher than that in the control samples (**Figure 5A**). Similarly, the expression levels of the seven genes were upregulated in atherosclerotic samples compared with control (GSE28829, **Figure 5B**), and *p*<0.05 was considered statistically significant. Then, we detected the expression levels of TYROBP, IL1B, C1QA, CCR5, C1QC, and MSR1 in thoracic aortas of mice. The results demonstrated that the expression of IL1B, C1QA, CCR5, and MSR1 was increased (*p*<0.05) in the model mice of T2DM complicated with AS compared with control groups (**Figure 5C**). Therefore, combining the above results, we identified four core genes, which are IL1B, C1QA, CCR5, and MSR1.

Subsequently, ROC curves were generated to further evaluate the diagnostic value of the validated core genes. The AUCs of IL1B, C1QA, CCR5, and MSR1 were 0.722, 0.778, 0.668, and 0.749, respectively, in the T2DM-related validation dataset (**Figure 5D**). Moreover, the AUC values of all validated core genes were >0.7 in the AS-related validation dataset, with AUC of 0.721, 0.938, 0.962, and 0.856 for IL1B, C1QA, CCR5, and MSR1, respectively (**Figure 5E**). At the multigene expression level, after linear fitting of all validated core gene expression models, the AUC value of the multigene combined diagnosis of T2DM and AS was 0.802 and 1.0, respectively (**Figures 5F, G**). These results reveal that core genes possess good discriminatory ability, and multigene combined diagnosis has a significantly higher predictive power than single gene.

### 3.5 Immune cell infiltration and correlation analysis

**Figure 6:**
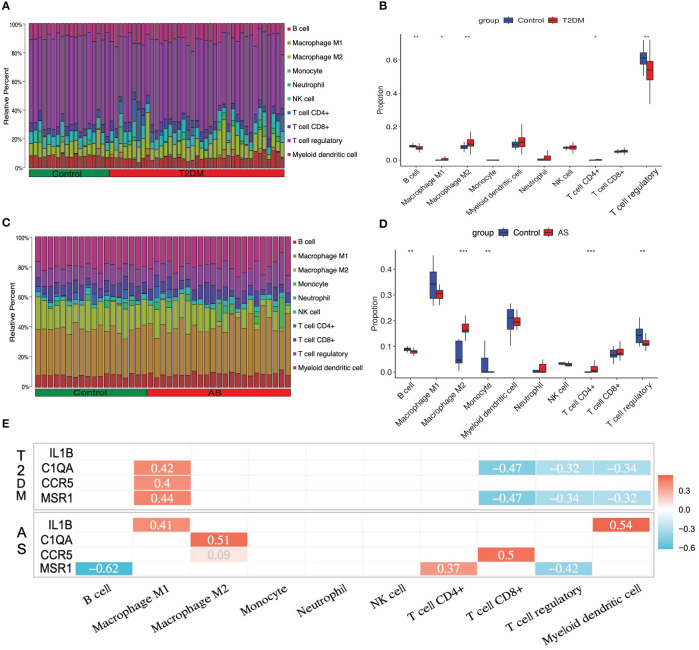
Immune cell infiltration analysis. The relative proportion of infiltrating immune cells (IICs) in **(A)** T2DM and **(C)** AS. Violin plot of distinct immune cell subtype compositions in T2DM vs. control **(B)** and AS vs. control **(D)**. **(E)** Correlation between core genes and immune infiltrating cells. Only statistically significant differences are reported in this heatmap. Red represents positive correlation, and blue represents negative correlation. *P < 0.05, **P < 0.01, ***P < 0.001.

As described above, the result of the enrichment analysis suggested that the immune response might play a crucial role in the course of T2DM complicated with AS. We examine how the immune system works by immune infiltration analyses. This analysis revealed that T2DM diseases were infiltrated by several immune cells, of which regulatory T cells (Tregs), myeloid dendritic cells, and M2 macrophages occupied the top 3 most counts of immune cell subpopulations (**Figure 6A**). Subsequently, we further analyzed differences in immune cell subgroups between the T2DM samples and the control pools (**Figure 6B**). The number of M1 and M2 macrophages and CD4+ T cells in the T2DM group was significantly higher than that in the control group (*p*<0.05 or *p*<0.01), while the number of B cells and Tregs was lower (*p*<0.01).

For the case of AS, the immune cell compositions of the AS and control group are shown in **Figure 6C**. M1 and M2 macrophages and myeloid dendritic cells occupied the top 3 most counts of immune cell subpopulations. Patients with AS had significantly higher numbers of M2 macrophages and CD4+ T cells than the controls (*p*<0.001), and there were fewer B cells, monocytes, and Tregs than those in the control group (*p*<0.01) (**Figure 6D**).

In order to investigate whether or not core genes were linked to IICs, the correlations were conducted using Spearman analysis. In T2DM, there is a positive relationship between C1QA, CCR5, MSR1, and M1 macrophages, and a negative relationship between C1QA, MSR1, and CD8+ T cells, Tregs, and myeloid dendritic cells (*p*<0.05 or *p*<0.01). In AS, there have a positive relationship between IL1B, C1QA, CCR5, MSR1, and M1 macrophages, M2 macrophages, CD4+ T cells, CD8+ T cells, and myeloid dendritic cells, and a negative relationship between MSR1 and B cells and Tregs (*p*<0.05 or *p*<0.01) (**Figure 6E**). The details of differential IICs selection results and correlation analysis are shown in **Supplementary Tables S12**, **S13**, respectively.

### 3.6 Integrated TF-mRNA network

**Figure 7:**
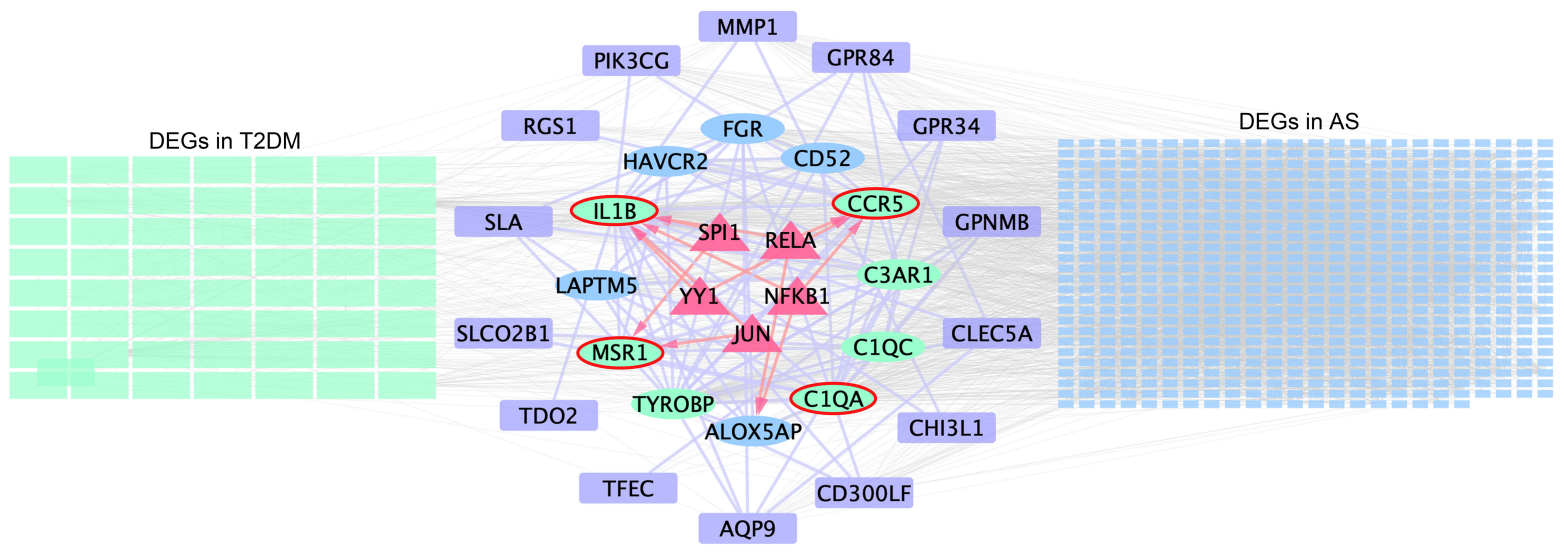
Schematic presentation of the TF-mRNA network. The green and blue rectangles on the left- and right-hand sides indicate the DEGs in T2DM and AS, respectively, and the common DEG analyses are shown in the middle. Ovals represent hub genes. Among them, genes included in the MCODE module are highlighted in green and core genes highlighted by red elliptical borders. Purple boxes indicate remaining genes in overlapping DEGs. Trigonal nodes in red represent key transcription factor genes regulating core gene expression.

TRRUST is a TF–target interaction database that shows regulatory regulation between TF and target genes. According to TF binding site information provided in TRRUST, potential key regulators for core genes were selected, a total of 10 associations between five TFs (SPI1, RELA, NFKB1, YY1, and JUN) and four core genes (IL1B, C1QA, CCR5, and MSR1). As shown in **Supplementary Table S14**, SPI1 regulated two genes (IL1B and MSR1), YY1 regulated two genes (IL1B and CCR5), JUN regulated two genes (IL1B and MSR1), RELA regulated two genes (IL1B and CCR5), and NFKB1 regulated two genes (CCR5 and IL1B). Based on this result, we constructed a regulatory TF-mRNAs network using Cytoscape software (**Figure 7**). We use different shapes or colors to distinguish different types of mRNA and TFs. This figure shows the potential pathological regulation process found in this study.

## 4 Discussion

Bioinformatics can help us better understand the complex biological processes. We identified 116 and 803 DEGs from the T2DM and AS datasets, respectively. Among these, 27 communal DEGs were identified between the two diseases. Based on enrichment analysis, these genes were significantly enriched in positive regulation of cytokine production, activation of immune response and negative regulation of leukocyte activation. Subsequently, 12 hub genes (TYROBP, IL1B, C1QA, C3AR1, FGR, CCR5, LAPTM5, C1QC, CD52, MSR1, HAVCR2, and ALOX5AP) were identified from four algorithms using the CytoHubba plugin. Then, MCODE was used to deconstruct the network for further dimension reduction. After external dataset and qRT-PCR experimental verification to these genes, a total of four core genes (IL1B, C1QA, CCR5, and MSR1) were obtained. ROC analysis indicated that those core genes owned higher diagnostic value both in T2DM and AS. Based on functional annotation analysis results, the immune system was emphasized as a critical component of T2DM complicated with AS. To further elucidate the potential biological roles of the core genes, we predicted their TFs in upstream and constructed a TF-mRNA regulatory network. Finally, based on CIBERSORT analysis, the correlation between key hub genes and IICs was evaluated to reveal the immune mechanism of T2DM and AS.

According to the immune infiltrating analysis, four differential immune cells (M2 macrophages, CD4+ T cells, B cells, and Tregs) regulated by C1QA, CCR5, and MSR1 may participate in the pathological process of T2DM complicated with AS. T2DM is a metabolic inflammatory disease mediated by a variety of immune cells and cytokines (17). In addition to innate immunity, such as macrophage and monocyte, adaptive immunity has also been confirmed to involved in this pathological process. CD4+T cells are an important subset of T cells, which are involved in the metabolic inflammation progressing through autocrine or paracrine. According to the functional characteristics of CD4+T cells, they can be divided into pro-inflammatory subsets (Th1, Th17, and Th22) and anti-inflammatory subsets (Tregs) (18). A proper balance between pro-inflammatory and anti-inflammatory subsets of CD4+T cells is essential for maintaining immune homeostasis and avoiding inflammatory response. The changes in the number and frequency of CD4+ Th subsets and the inflammatory response produced by cytokines are related to T2DM (19). Instead, Treg is a protective subtype of CD4 cells, which is related to its effect on macrophage exocytosis promoting and plaque remodeling (20, 21). The results of this study showed that CD4+T cells and Tregs showed an upward and downward trend in both two datasets, respectively.

B cells contribute significantly to innate and adaptive immunity by producing antibodies and cytokines (22, 23). B1 cell is an important subtype of B cell. It can synthesize and release IgM, a natural antioxidant low-density lipoprotein, which inhibits the uptake of ox-LDL by macrophages and ultimately inhibits the production of foam cells. In addition, the IgM can also inhibit the formation of necrotic core in AS plaque (24). However, the related research on this cell is rarely reported in the field of diabetes. In this study, the relative proportions of B cells showed significant decrease in two diseases, respectively. In addition to B cells and T cells, the changes in the number of macrophages show multiplicity changes. Different from the single view that M1 macrophages are involved in pro-inflammatory responses and M2 macrophages are involved in anti-inflammatory responses (25), the results of this study show that the relative proportion of M1 and M2 macrophages in T2DM is increased, while that of M2 macrophages in AS is significantly decreased. This exhibits that the macrophages have a high degree of plasticity in response to microenvironmental stimulus.

Complement protein C1q is a complex glycoprotein component of the classical complement pathway with 18 polypeptide chains. C1QA is among the three genes encoding C1q and is crucial in the innate immune response (26). C1q is significantly higher in advanced atherosclerotic plaques and those in patients with acute coronary syndrome than in early lesions and those with stable angina pectoris (27). In T2DM, C1QA protein abundance is altered in patient serum (28). Conversely, C1q has pro- and atheroprotective effects; however, few studies have focused on its role in T2DM (29). These results indicate that C1q is involved in the progression of AS and T2DM.

Some studies have shown that MSR1 polymorphisms are associated with AS and plasma fatty acid distribution (30). It is a scavenger receptor and can promote macrophage inflammation (31, 32). MSR1 was distributed in the macrophages and smooth muscle cells (33, 34), and its induction of atherosclerotic lesion formation facilitates phagocytosis (35, 36). Previous studies have demonstrated that high MSR1 expression causes cholesterol to feed into the vessel wall, whereas its deficiency causes a reduction in spontaneously developed AS (37). Advanced glycation end products are crucial in diabetes. Under high-advanced glycation end-product intake, MSR1 expression showed a tendency towards with insulin levels and may promote endocrine-related diseases (38).

Our enrichment results suggest that positive regulation of cytokine production and signaling in the immune system may regulate diseases. RELA (p65) and NFκB1 (p50) belong to the NFκB family. Both contain a Rel homologous domain at the N-terminal, which can mediate the specific binding, dimerization, and binding of NFκB to DNA. RELA and NFκB1 can also combine and form homologous or heterodimers. The binding of different dimers to DNA has different effects on inflammation regulation. The binding of p50/p50 and p65/p65 homodimers to DNA suppresses the expression of inflammatory genes, while p50/p65 heterodimer promotes the expression of pro-inflammatory factors related to NFκB (39, 40). Activating NFκB finally induces the synthesis and release of cytokines, such as TNF-α and IL-1β, and affects ROS levels, which can directly stimulate islet β-cell apoptosis and cause damage by activating macrophage and T-cell attack on islet β cells (41). In addition, NFκB can cause vascular endothelial injury, vascular smooth muscle proliferation, and foam cell formation (42).

The JUN family includes c-Jun, Jun B, and Jun D, which are the downstream proteins of the primary functions of the JNK signaling pathway. C-Jun exacerbates atherogenesis by decreasing cholesterol efflux from macrophages in atherosclerotic plaques (43). c-Jun is a novel regulator of T-cell lineage development and decision-making (44). In T2DM, the activation of JNK directly phosphorylates insulin receptor substrate 1, producing ROS and impairing insulin signaling. The active ASK1 induces pancreatic β-cell death (45). Emerging evidence suggests that JNK is involved in regulating cellular senescence by downregulating hypoxia-inducible factor-1α to accelerate hypoxia (46). This may be associated with the progression of T2DM. The SPI-1 proto-oncogene (SPI1) is crucial in the hematopoietic system, normal and pathogenic (47). SPI1 upregulation reportedly stimulated the TLR4/NFκB axis and aggravated myocardial infarction (48). Further findings suggested that SPI1 regulates copper homeostasis in diabetic cardiomyopathy (49). However, experimental evidence of SPI1 expression is lacking.

Various innate and adaptive immune cells promote the formation of an inflammatory microenvironment and are crucial in the progression of AS. Low-grade inflammation, essential for AS development, is an important feature of diabetes (50). The development of diabetic AS induces an immune microenvironment that shifts the normal balance toward a pro-inflammatory state. Although the immune microenvironment remains investigated, its exact role remains unknown. We hypothesized that AS and T2DM share a common pathogenesis correlated with B cells, CD4+ T cells, Tregs, and M2 macrophages. Our results reveal that the relationship among five TFs, four core genes, and four immune cells profile may be crucial in understanding the pathogenesis therapeutic direction of T2DM complicated with AS. This represents a promising avenue to treat and prevent diseases. This study focuses on the common mechanisms and identification of hub genes and immune infiltration profiles in patients with AS and T2DM.

However, there are a few limitations to this study. First, despite the large sample size and experimental verification, it is a retrospective study that requires validation through a prospective study. Second, although validated in animal models, these core genes have not been evaluated in humans. Third, specific molecular mechanisms of immune responses regulated by core genes in T2DM complicated with AS remain poorly determined. These will be the focus of our future studies.

## 5 Conclusion

We identified four core genes (IL1B, C1QA, CCR5, and MSR1) and four diff-IICs (B cells, CD4+ T cells, Tregs, and M2 macrophages). The evidence of common pathogenesis points toward the immune microenvironment after core genes modulation, which might be modulated by five TFs (RELA, NFκB1, JUN, YY1, and SPI1). These results provide a direction for future studies on the potential key genes in patients with T2DM complicated with AS.

## Data availability statement

The datasets presented in our study are available from the online repositories. Detailed information about it can be found in the article.

## Ethics statement

Ethical approval was not required for the study involving humans in accordance with the local legislation and institutional requirements. Written informed consent to participate in this study was not required from the participants or the participants’ legal guardians/next of kin in accordance with the national legislation and the institutional requirements. The animal study was approved by animal ethics committee of the Ethics Committee of Xiyuan Hospital, China Academy of Chinese Medical Sciences. The study was conducted in accordance with the local legislation and institutional requirements.

## Author contributions

YQ: Writing – review & editing, Writing – original draft, Investigation, Data curation, Conceptualization. YaZ: Writing – original draft, Software, Investigation, Data curation. SG: Writing – original draft, Methodology, Formal analysis, Data curation. LL: Writing – original draft, Methodology, Formal analysis, Data curation. HW: Writing – original draft, Visualization, Validation, Formal analysis. YC: Writing – original draft, Visualization, Validation, Formal analysis. QZ: Writing – original draft, Visualization, Validation, Methodology, Formal analysis. YiZ: Writing – review & editing, Supervision, Resources, Project administration, Funding acquisition. FX: Writing – review & editing, Supervision, Resources, Project administration.

## Supplementary material

Supplementary Figure 1 The hub genes and their co-expression genes analyzed using GeneMANIA.
