# Supplementary figures and images for: Common ground on immune infiltration landscape and diagnostic biomarkers in diabetes-complicated atherosclerosis: an integrated bioinformatics analysis
Source: Front Endocrinol (Lausanne). 2024 Jul 31;15:1381229. doi: 10.3389/fendo.2024.1381229 (PMC11323117; doi:10.3389/fendo.2024.1381229)

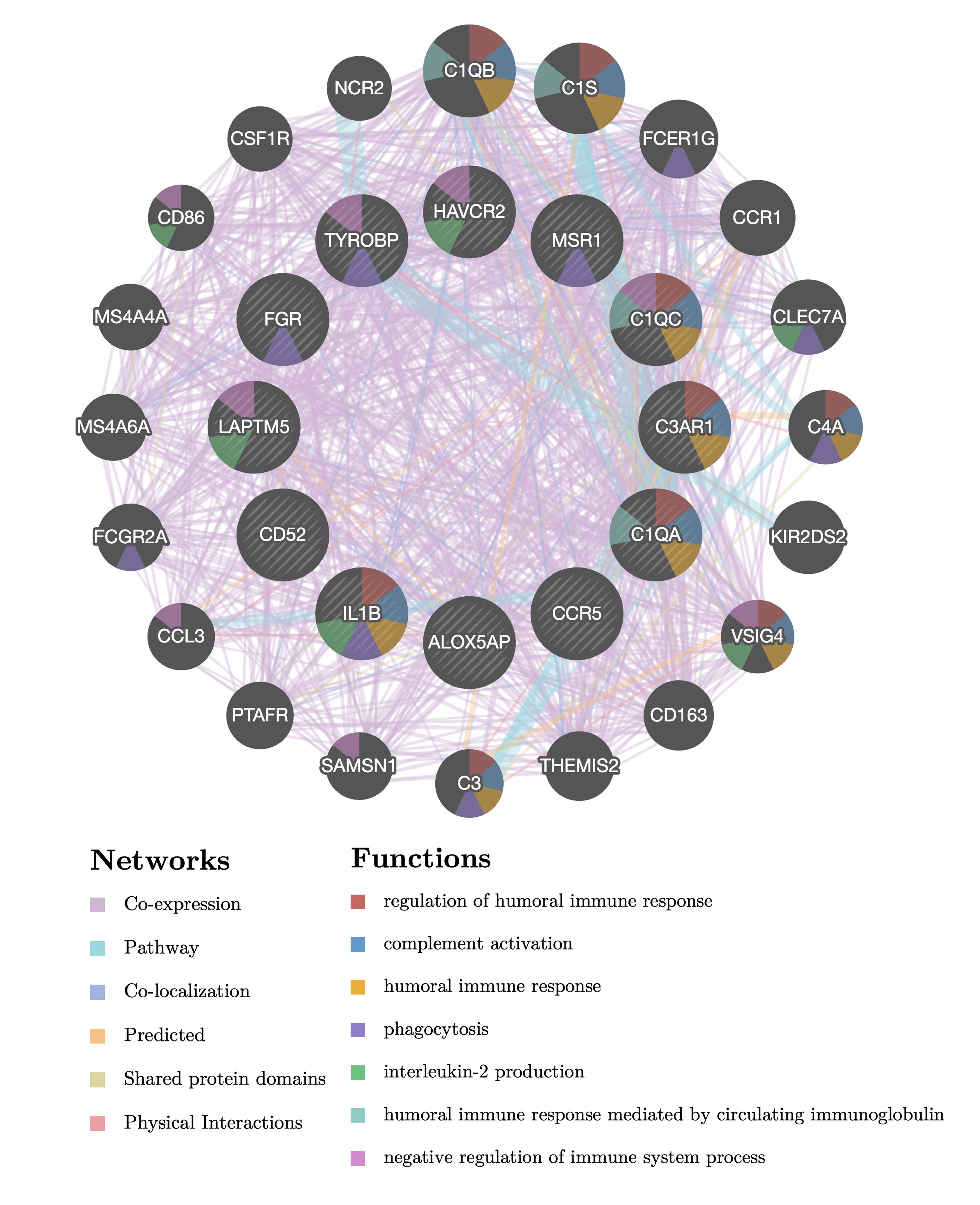

Supplement: Supplementary Figure 1 — The hub genes and their co-expression genes analyzed using GeneMANIA. [file Image_1.jpg]
